# Group 1 innate lymphoid cells and inflammatory macrophages exacerbate fibrosis in creeping fat through IFN-γ secretion

**DOI:** 10.1007/s00535-025-02243-x

**Published:** 2025-03-29

**Authors:** Ryota Mori, Takayuki Ogino, Mari Murakami, Hisako Kayama, Daisuke Okuzaki, Atsuyo Ikeda, Yuki Sekido, Tsuyoshi Hata, Atsushi Hamabe, Hidekazu Takahashi, Norikatsu Miyoshi, Mamoru Uemura, Hiroki Ikeuchi, Kiyoshi Takeda, Tsunekazu Mizushima, Yuichiro Doki, Hidetoshi Eguchi

**Affiliations:** 1 Department of Gastroenterological Surgery, Graduate School of Medicine, Osaka University, 2-2 Yamadaoka, Suita, Osaka 565-0871 Japan; 2 Department of Therapeutics for Inflammatory Bowel Diseases, Graduate School of Medicine, Osaka University, 2-2 Yamadaoka, Suita, Osaka 565-0871 Japan; 3 Immunology Frontier Research Center, Osaka University, Osaka, Japan; 4 Laboratory of Immune Regulation, Department of Microbiology and Immunology, Graduate School of Medicine, Osaka University, Osaka, Japan; 5 Institute for Advanced Co-Creation Studies, Osaka University, Osaka, Japan; 6 Genome Information Research Center, Research Institute for Microbial Diseases, Osaka University, Osaka, Japan; 7 Laboratory of Innate Immunity, Department of Microbiology, Infectious Diseases and Immunology, Charité-Universitätsmedizin Berlin, Berlin, Germany; 8 Department of Surgery, Dokkyo Medical University, Tochigi, Japan; 9 Division of Inflammatory Bowel Disease Surgery, Department of Gastroenterological Surgery, Hyogo Medical University, Hyogo, Japan

**Keywords:** Crohn's disease, Mesenteric fat, Fibrogenesis, Group 1 Innate lymphoid cell

## Abstract

**Background:**

Creeping fat is a characteristic of Crohn's disease and impacts the disease course. We evaluated creeping fat formation, focusing on innate lymphoid cell-mediated fibrogenesis and its clinical significance.

**Methods:**

Patients with inflammatory lesions in the ileum (the most commonly affected area), who underwent surgical resection at Osaka University or Hyogo Medical University (*n* = 34), were included. The ileum and mesentery were obtained from three sites: the control, non-creeping fat part, and creeping fat part. The distribution and properties of the innate lymphoid cells were analyzed by cell isolation. Furthermore, the correlation between macrophages and their effects on adipose tissue and clinical course were also investigated in a prospective cohort study.

**Results:**

Group 1 innate lymphoid cells in creeping fat were increased, correlating with inflammatory macrophages in the mesentery and showing higher interferon-γ expression. Co-culture experiment involving stromal vascular fraction from the control mesentery and Group 1 innate lymphoid cells from creeping fat revealed increased mRNA expression of fibrosis-related genes and inflammatory markers of macrophages, although these changes were nullified by interferon-γ-neutralizing antibody. Next, we examined the clinical importance of Group 1 innate lymphoid cells and identified their high frequency in creeping fat as a risk factor for early recurrence (*P* = 0.008, odds ratio: 1.19). Furthermore, the higher Group 1 innate lymphoid cell frequency group (≥ 80%) had shorter relapse-free survival (*P* = 0.03).

**Conclusions:**

Group 1 innate lymphoid cells and inflammatory macrophages contribute to creeping fat formation via interferon-γ secretion, affecting post-surgery intestinal outcomes.

**Supplementary Information:**

The online version contains supplementary material available at 10.1007/s00535-025-02243-x.

## Introduction

Crohn's disease (CD) is a chronic inflammatory disease of the gastrointestinal tract that progresses in relapsing and remitting manners, particularly in the terminal ileum [1]. A notable characteristic of the disease is an endoscopically visible wrapping of mesenteric fat, also known as “creeping fat (CrF)”, around the inflamed and fibrotic intestine. Most patients with CD develop fibrosis-induced intestinal obstruction through the disease course [2]. Most of them eventually require surgery, and repeated surgery is also not rare. From a clinicopathological perspective, CrF is associated with obstruction resulting from changes in the connective tissue of the intestinal wall via adipose tissue encroachment into the intestinal muscularis.

CrF was first reported by Dr. Burrill B. Crohn in the early 1930s as a mesenteric abnormality associated with CD; however, its pathogenesis has not been clarified ever since [3]. Curiously, CrF is not seen in ulcerative colitis, which also causes inflammation in the intestinal tract [4]. Our previous study, which analyzed pathological images of resected tissue from patients with CD using artificial intelligence, showed that adipocyte shrinkage in the mesentery was a predictor of recurrence [5]. Moreover, magnetic resonance imaging revealed that alterations in the mesentery occur before ulcers in the mucosa can be observed by colonoscopy [6]. These results suggest that CrF is a key factor in CD pathogenesis and activity. A recent study reported that CrF formation is promoted by gut microbiota; however, the detailed mechanism is unknown [7]. The elucidation of these mechanisms could lead to improvements in the treatment of fibrosis-induced stenosis.

Traditionally, visceral fat was considered to play a passive role in the body as a form of storage for excess calories and a conduit for blood and lymphatic vessels and the enteric nervous systems [8]. However, it has become clear that fat is not a passive tissue, but constitutes a complex environment involving many different cell clusters that influence physiological processes [9, 10]. Meanwhile, innate lymphoid cells (ILCs) are tissue-resident lymphocytes that lack adaptive antigen receptors and are classified as ILC1s, ILC2s, ILC3s, natural killer cells, and lymphoid tissue inducer cells. ILCs are abundant in organs such as the lungs, intestinal tract, and adipose tissue, and are involved in tissue homeostasis including morphogenesis, metabolism, regeneration, and growth [11]. ILC1s are a main source of pro-inflammatory cytokines, including interferon (IFN)-γ and tumor necrosis factor (TNF)-α. A recent study showed that in high-fat-fed mice, ILC1s were accumulated in subcutaneous adipose tissue and directly induced fibrogenesis and inflammation together with inflammatory macrophages [12]. Therefore, we hypothesized that ILCs contributed to fibrogenesis with CrF formation.

The aim of this study was to evaluate the functional mechanisms and formation of CrF, focusing on fibrogenesis mediated by ILCs. Additionally, we demonstrated the clinical significance of ILC1 in CrF.

## Materials and methods

### Ethics

This study was approved by the ethical committees of Osaka University School of Medicine (permit number: 10261) and Hyogo Medical University (permit number: 0407). Written informed consent was obtained from all patients for the use of their samples and data.

### Tissue samples

The CD sample included patients with inflammatory lesions in the ileum, which is the most frequently affected site. The patients underwent resection of the inflamed portion at Osaka University or Hyogo Medical University. The mesentery was resected near the intestinal tract.

**Fig. 1:**
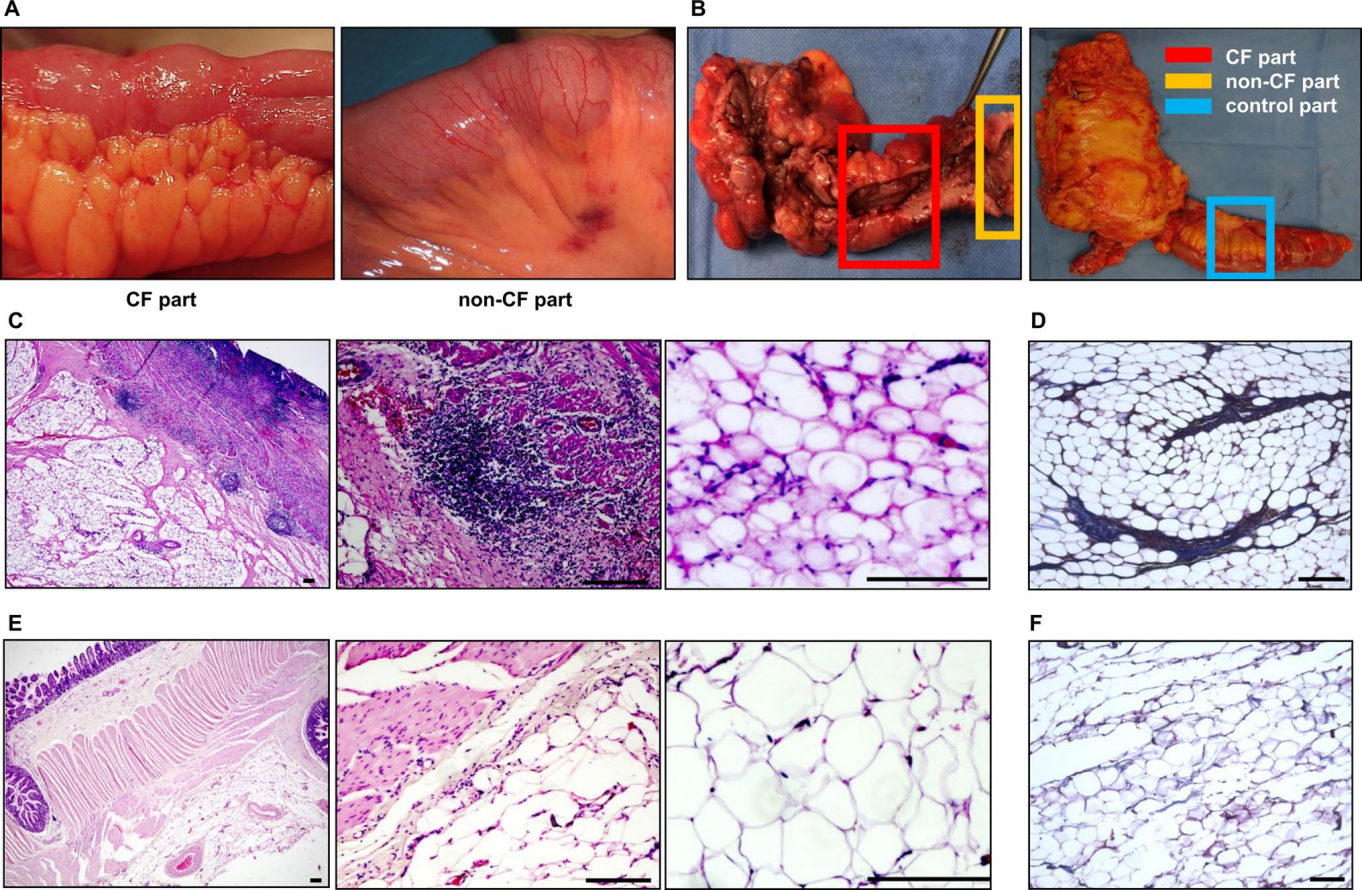
Comparison of magnified and stained surgical specimens of CrF and control mesentery. **A** Magnified surgical specimen: creeping fat (CrF) part (left), non-CrF part (right). **B** Surgical specimen: Crohn’s disease (left), control (right). Normal ileum and mesentery were obtained from patients with colorectal cancer as control samples. Areas encircled in red were the CrF part, in orange the non-CrF part, and in blue the control part. **C** Representative images of hematoxylin and eosin (H&E) staining of the CrF part: overview of the intestine and mesentery (left), intestinal attachment area of the mesentery (middle), and the mesentery (right). **D** Representative images of H&E staining of the control part: overview of the intestine and mesentery (left), intestinal attachment area of the mesentery (middle) and the mesentery (right). **E** Representative image of Masson trichrome staining of creeping fat. **F** Representative image of Masson trichrome staining of the control mesentery. Scale bars, 200 µm

Areas showing CrF with macroscopically strong inflammation were defined as “CrF parts” (Fig. 1A; left), and areas with slight or no inflammation near the transected edges were defined as “non-CrF parts” (Fig. 1A; right).

The ileum and mesentery were obtained from the CrF and non-CrF parts in pairs (Fig. 1B, left).

Normal ileum and mesentery were obtained from 15 patients with colorectal cancer as control samples. They were collected in pairs from macroscopically non-inflamed areas sufficiently distant from the tumors (Fig. 1B; right). These patients underwent intestinal resection for cancer at Osaka University. Patients with autoimmune diseases, who received chemoradiation therapy before surgery, or who underwent emergency surgery for perforation or ileus, were excluded from the study.

All tissues were obtained immediately after surgical resection, and the experiments were subsequently started. Patient characteristics are provided in Supplementary Table 1.

### Cell isolation

Human stromal vascular fractions (SVFs) and lamina propria cells (LPCs) were isolated using a previously described protocol with partial modifications [13, 14]. Briefly, both the mucosa and mesentery were washed in PBS to remove feces and blood, and then weighed. Mucosal samples were placed in Hank's balanced salt solution containing 5 mmol/L ethylenediaminetetraacetic acid and incubated for 5 min with shaking at room temperature. After washing with phosphate-buffered saline (PBS), both tissue samples were minced mechanically into small pieces, then enzymatically digested with 1 mg/mL collagenase II (Worthington Biochemical Corporation, NJ, USA) and 80 U/mL DNase I (Sigma-Aldrich, MO, USA) in RPMI1640 medium containing 4% fetal bovine serum (FBS) for 60 min in a 37 °C shaking water bath. The obtained tissues were filtered through a 40 mm cell strainer (Corning Inc., NY, USA), followed by the addition of ammonium chloride–potassium lysis buffer and shaking at 37 °C for 5 min to remove the erythrocyte component. Subsequently, centrifugation was performed to collect the LPCs and SVFs, which were then washed with PBS containing 2% FBS.

### Flow cytometry

Isolated cells were stained with surface antibodies for 30 min at 4 °C. For intranuclear transcription factor staining, a Foxp3/Transcription Factor Staining Buffer Kit (eBioscience, CA, USA) was used according to the manufacturer's instructions. For the staining of intracellular cytokines after stimulations, cells were stained by surface markers for 20 min at 4 °C. The cells were then treated with Cytofix/Cytoperm (BD Biosciences, CA, USA) for 20 min at 4 °C. Next, Perm/Wash Buffer (BD Biosciences) was added with antibodies against cytokines.

Flow cytometric analysis and cell sorting were performed using FACSAria II (BD Biosciences), and the data were analyzed using FlowJo Software (FlowJo LLC, OR, USA).

The anti-human antibodies used are listed in Supplementary Table 2.

### Morphological analysis

Isolated ILCs were spread on glass slides using a cytospin (Thermo Shandon, PA, USA), air-dried, and fixed with methanol for 5 min. After air-drying, the cells were stained with Giemsa stain for 30 min at room temperature. The stained slides were rinsed with deionized water, air-dried, and morphologically evaluated. All images were captured using BIOREVO BZ-X800 (Keyence, Osaka, Japan).

### RNA extraction and quantitative PCR

Total RNA was extracted using the GenElute Mammalian Total RNA Miniprep Kit (Sigma-Aldrich) and cDNAs was generated using the ReverTra Ace qPCR RT Master Mix with gDNA Remover (Toyobo, Osaka, Japan). qRT-PCR was performed on a StepOnePlus Real-Time PCR System (Applied Biosystems, MA, USA) using Power SYBR Green PCR Master Mix (Applied Biosystems). The amplification conditions were 95 °C for 10 min, followed by 45 cycles of 95 °C for 15 s and 60 °C for 1 min. All data were normalized to the expression of GAPDH and presented as relative expression using the ΔΔC_T_ method. Primer sets are listed in Supplementary Table 3. The RNA sequencing method is described in Supplementary Data.

### Histological examination

Tissue samples were fixed in 10% formalin and care was taken not to introduce deformities. After dehydration with an ethanol concentration series, samples were embedded in paraffin and the resulting blocks were sectioned at 3 µm onto slides. The sections were stained with hematoxylin and eosin or Masson’s trichrome (Cosmo Bio, Tokyo, Japan). All images were captured using BIOREVO BZ-X800 (Keyence).

### Immunohistochemistry

For immunohistochemistry, paraffin blocks were deparaffinized and sectioned at 3 µm onto slides. Each slide was boiled for 15 min in 10 mmol/L citrate buffer (pH 6) for antigen retrieval and then immersed in a methanol–hydrogen peroxide solution for 30 min at room temperature to block peroxidase activity. Then, the slides were blocked with blocking serum for 30 min at room temperature and incubated overnight with the primary antibody at 4 °C, followed by a 30 min incubation with the secondary antibody at room temperature. For double staining, MACH2 Double Stain 2 (Biocare Medical, CA, USA) was used as the secondary antibody. Peroxidase staining was visualized with the Betazoid DAB Kit (Biocare Medical), and alkaline phosphatase staining with Vulcan Fast Red (Biocare Medical). Finally, the slides were counterstained with hematoxylin, dehydrated with graded alcohol and xylene, and mounted.

The following primary antibodies were used: rabbit anti-CD3 (1:100, AB16669-100, Abcam, Cambridge, UK), mouse anti-T-bet (1:100, sc-21749; Santa Cruz Biotechnology, CA, USA), and rabbit anti-CD68 (1:200, D4B9C; Cell Signaling Technology, MA, USA). All images were captured using BIOREVO BZ-X800 (Keyence).

### Cell stimulation

ILC1s were activated using phorbol 12-myristate 13-acetate (10 ng/mL) and ionomycin (250 ng/mL) and incubated at 37 °C for 2 h. For cytokine detection, monensin (GolgiStop; BD Pharmingen, CA, USA) was added, and the cells were incubated for 2 h.

### Cell co-culture

Viable ILC1s and SVFs were isolated from 15 g of CrF and cultured in the upper chamber of the Transwell inserts in a 12-well plate. On the other hand, 1 × 10^6^ of SVFs isolated from the control mesentery were seeded in the lower chamber and co-cultured at 37 °C and 5% CO_2_. RPMI medium supplemented with 10% FBS, 55 mM 2-mercaptoethanol, 100 U/mL penicillin/streptomycin, 50 ng/mL IL12 (PeproTech EC Ltd, London, England), 100 ng/mL IL18 (PeproTech), 5 μg/mL trehalose-6,6’-dimycolate (Nacalai Tesque, Kyoto, Japan), and 200 μM palmitate (Sigma-Aldrich) was used to mimic the in vivo microenvironment. The gene expression in control SVFs was analyzed after 72 h.

### Postoperative endoscopic examination and definition of postoperative recurrence

Patients with Crohn's disease were also studied for postoperative endoscopic recurrence as a prospective cohort study after analysis of surgical specimens. Postoperative endoscopic examinations of the intestinal tract were performed approximately 6 months after surgery. The frequency of subsequent examinations was determined by physicians, considering the patient's condition. The Rutgeerts score was used as an indicator of postoperative endoscopic recurrence [15]. Postoperative recurrence was defined as a Rutgeerts score of i2 or higher.

### Statistical analysis

Statistical analyses were performed using GraphPad Prism 9.0 (GraphPad Software) or JMP pro 14.0.0 (SAS Institute Inc.). All data are presented as mean ± standard error of the mean (SEM). Categorical variables were compared using the chi-squared test. For comparisons between two groups with normal distribution and homoscedasticity, paired t tests were used if there was correspondence, otherwise t tests were used. For comparisons between two groups with non-normal distribution and heteroscedasticity, the Wilcoxon signed-rank test was used if there was correspondence, otherwise, the Mann–Whitney U test was used. Correlations between two groups were determined using Pearson's simple linear regression analysis. A *P* value lower than 0.05 was considered statistically significant.

## Results

### Activation of immune response and severe fibrosis in CrF

First, we examined the genetic and pathological features of CrF. Comprehensive genomic analysis using the K-means clustering method revealed a set of genes, known as Cluster A genes, expressed in the mesentery of patients with CD. These included immune response, lymphocyte activation, and immune regulatory genes, suggesting that the immune response was highly activated (Supplementary Fig. 1A; Table 1).

**Table 1:** Results of RNA sequencing analysis

Cluster	Adjusted *P* values	nGenes	Pathways
A	3.2e-16	64	Adaptive immune response
	3.2e-16	143	Regulation of immune system process
	3.2e-16	84	Lymphocyte activation
	4.3e-16	219	Immune system process
	1.5e-15	173	Immune response
	1.6e-12	61	T cell activation
	2.5e-12	121	Cell activation
	1.9e-11	109	Leukocyte activation
	2.1e-11	40	Antigen receptor-mediated signaling pathway
	2.5e-11	89	Positive regulation of immune system process
	5.7e-11	64	Regulation of cell activation
	5.7e-11	55	Regulation of lymphocyte activation
	4.4e-10	45	Regulation of T cell activation
	5.8e-10	59	Regulation of leukocyte activation
	6.1e-09	45	Immune response-activating cell surface receptor signaling pathway
B	5.9e-05	4	Triglyceride catabolic process
	5.9e-05	4	Neutral lipid catabolic process
	5.9e-05	4	Acylglycerol catabolic process
	8.7e-05	5	Digestion
	2.2e-04	4	Glycerolipid catabolic process
	3.9e-04	2	Negative regulation of very-low-density lipoprotein particle remodeling
	5.3e-04	4	Retinoid metabolic process
	5.3e-04	4	Triglyceride metabolic process
	5.9e-04	4	Diterpenoid metabolic process
	7.8e-04	4	Terpenoid metabolic process
	9.5e-04	4	Acylglycerol metabolic process
	9.6e-04	4	Isoprenoid metabolic process
	9.6e-04	2	Positive regulation of guanylate cyclase activity
	9.6e-04	2	Regulation of Cdc42 protein signal transduction
	9.6e-04	4	Positive regulation of small molecule metabolic process
C	6.6e-14	46	Defense response
	6.6e-14	59	Response to external stimulus
	1.2e-13	33	Inflammatory response
	6.2e-13	69	Response to stress
	2.3e-12	50	Immune response
	4.5e-12	61	Response to organic substance
	5.7e-12	41	Biological process involved in interspecies interaction between organisms
	5.7e-12	35	Cellular response to cytokine stimulus
	7.4e-12	36	Response to cytokine
	8.0e-11	37	Response to external biotic stimulus
	8.0e-11	37	Response to other organism
	8.5e-11	56	Immune system process
	2.8e-10	40	Response to oxygen-containing compound
	4.3e-10	51	Cellular response to organic substance
	6.9e-10	34	Positive regulation of developmental process
D	4.8e-18	248	Cellular response to chemical stimulus
	1.2e-16	232	Immune system process
	1.4e-15	241	Response to organic substance
	1.4e-15	120	Response to cytokine
	1.5e-13	202	Cellular response to organic substance
	3.3e-13	267	Response to stress
	3.3e-13	142	Defense response
	5.7e-13	173	Immune response
	5.1e-12	105	Cellular response to cytokine stimulus
	5.5e-12	200	Response to external stimulus
	9.6e-12	136	Regulation of immune system process
	1.7e-11	83	Cytokine-mediated signaling pathway
	6.0e-11	84	Inflammatory response
	1.2e-10	65	Response to bacterium
	1.4e-09	258	Regulation of response to stimulus

The *P*-value represents the probability of significance following multiple testing correction in the path analysis. A p-value less than 0.05 indicates that the path is considered ‘characteristic for that cluster’

Next, we investigated the histopathological features of CrF compared to those of the control. In CrFs, cell aggregation and infiltration were observed in the intestinal wall, breaking through the serosa (Fig. 1C; left and middle panels). In addition, the connective tissue within the adipose tissue was thickened due to surrounding cell clusters (Fig. 1C; right). On the other hand, in the control mesentery, only a small number of cells were found, with no evidence of adipose tissue wall thickening or cell infiltration into the mesentery (Fig. 1D). Adipocytes in CrF were round and smaller in size compared to those in the control mesentery (66.9 ± 2.1 µm vs 156.3 ± 9.5 µm; *P* < 0.0001; Supplementary Fig. 1B). Masson’s trichrome staining revealed substantial fibrosis in CrF, particularly in areas of adipose tissue wall thickening; however, fibrosis was rarely observed in the control mesentery (Fig. 1E-F). RNA sequence analysis demonstrated that the expression of representative fibrosis-related genes and extracellular matrix (ECM) regulators, including *COL1A*, *COL3A*, *ACTA2*, and *PDGFB*, increased in CD (Supplementary Fig. 1C). These data suggest that inflammation and significant fibrosis occur in CrF.

### Characterization of human ILC subsets in mesentery of ileum

Next, we examined the presence of ILCs in human adipose tissue from the subcutaneous fat, mesentery, and omentum of patients who underwent surgery for colorectal cancer. The frequency of the ILCs varied depending on the organ (Supplementary Fig. 1D). More than 60% of patients with CD have inflammatory lesions in the ileum [16]; therefore, we focused on the mesentery of the ileum in our subsequent analyses.

**Fig. 2:**
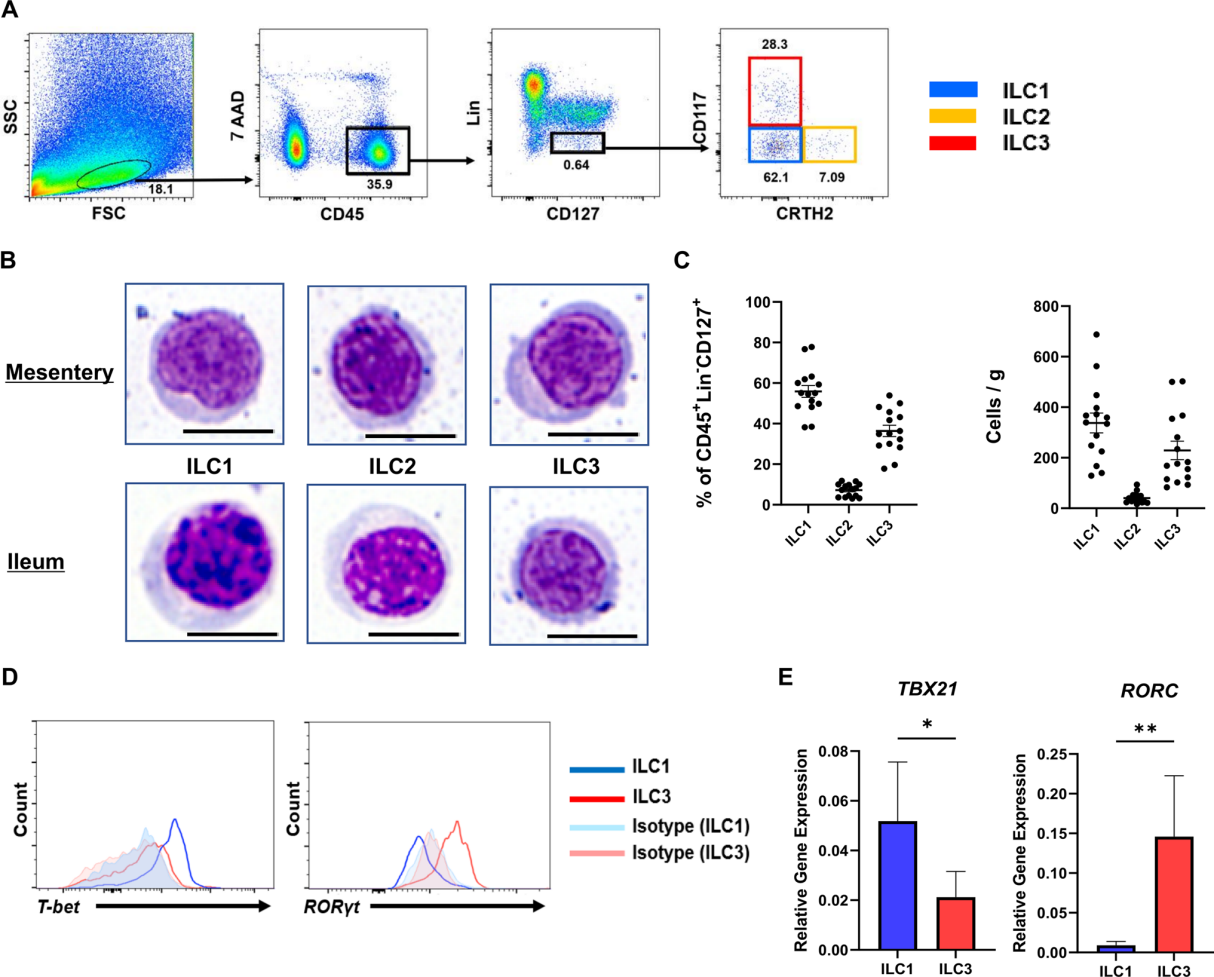
Characterization and analysis of ILC subsets in the human mesentery. **A** Gating of ILC1, ILC2, and ILC3 from human mesentery by flow cytometry. Among 7AAD^−^ CD45^+^ Lin^−^ (CD3^−^ CD11c^−^ CD14^−^ CD16^−^ CD19^−^ CD20^−^) CD127^+^ cells, CD117^−^ CRTH2^−^ cells were defined as ILC1, CD117^−^ CRTH2^+^ as ILC2, and CD117^+^ CRTH2^−^ cells as ILC3. **B** Morphological analysis of ILC subsets from mesentery and ileum through May–Giemsa staining. Scale bars, 10 µm. **C** Frequencies and absolute cell number/g (tissue weight) of the ILC subsets among 7AAD^−^ CD45^+^ Lin^−^ (CD3^−^ CD11c^−^ CD14^−^ CD16^−^ CD19^−^ CD20^−^) CD127^+^ cells in the control mesentery. **D** Flow cytometric analysis of T-bet and RORγt expression in ILC subsets purified from the ileal mesentery. Representative data of seven samples each. [E] Expression of *TBX21* and *RORC* in ILC subsets purified from CrF by qPCR. Data are presented as means ± standard error of the mean (SEM) of seven independent donors each (*P < 0.05, **P < 0.01). Representative data from subcutaneous fat, omentum, and mesentery of the colon are shown

In the ileal mesentery analysis, CD45^+^ Lin^−^ (CD3^−^ CD11c^−^ CD14^−^ CD16^−^ CD19^−^ CD20^−^) CD127^+^ cells among SVFs were divided into CD117^−^ CRTH2^−^ (ILC1s), CD117^−^ CRTH2^+^ (ILC2s), and CD117^+^ CRTH2^−^ (ILC3s) cells using flow cytometry (Fig. 2A). The three ILC subsets from the control mesentery exhibited lymphoid morphologies similar to those of ILCs from ileal LPCs, as determined by May–Giemsa staining (Fig. 2B). Fluorescence-activated cell sorting analysis of the control mesentery revealed exceedingly small populations of ILC2s (Fig. 2C). ILC1s exhibited high expression of T-bet, a master regulator of ILC1 encoded by *TBX21*, whereas ILC3s showed high expression of RORγt, which is encoded by *RORC* and responsible for the development of ILC3s (Fig. 2D–E). These results showed that ILCs are present in relatively small amounts in human fat tissue but can be firmly classified.

### Distribution of ILCs in CrF and correlation with ILCs in the ileum

**Fig. 3:**
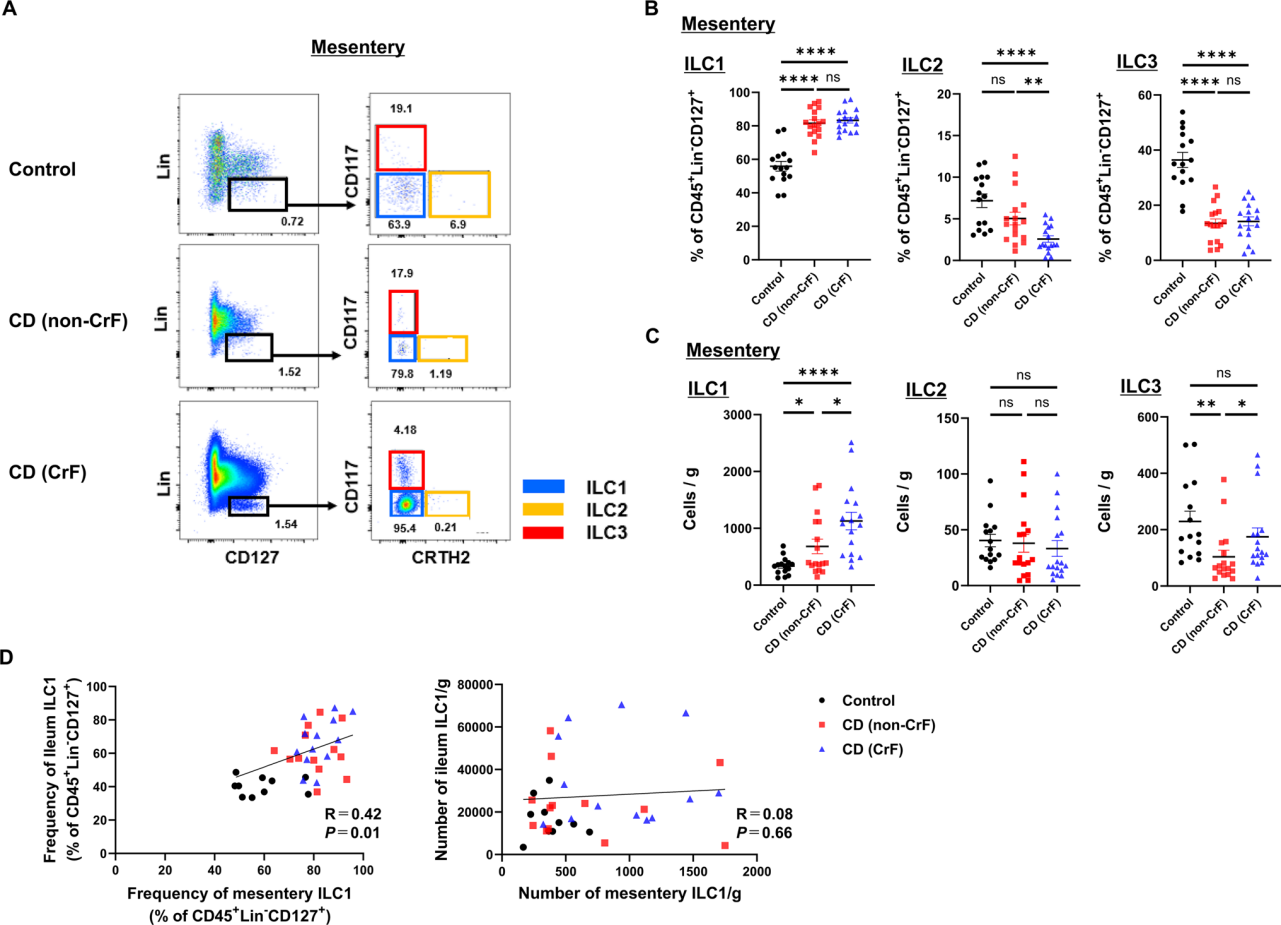
Correlation between ILC subsets in the human mesentery and inflammation. **A** Flow cytometric analysis of ILC subsets from human mesentery. Representative data from the control, CD (non-CrF), and CD (CrF) are shown. **B** Frequencies of ILC subsets among 7AAD^−^ CD45^+^ Lin^−^ (CD3^−^ CD11c^−^ CD14^−^ CD16^−^ CD19^−^ CD20^−^) CD127^+^ cells depending on the state of inflammation in human mesentery. **C** Absolute cell number/g (tissue weight) of ILC subsets depending on the state of inflammation in the mesentery. Data are presented as mean ± SEM of 15 control donors and 17 donors with Crohn's disease (*ns* not significant; *P < 0.05, **P < 0.01, ****P < 0.0001). **D** Correlation of the frequency and absolute cell number/g (tissue weight) of ILC1s among 7AAD^−^ CD45^+^ Lin^−^ (CD3^−^ CD11c^−^ CD14^−^ CD16^−^ CD19^−^ CD20^−^) CD127^+^ cells between ileum and mesentery. *P* values were obtained through Pearson's simple linear regression analysis

Next, we examined how ILC subsets are altered in the CrF and whether they correlate with the ILCs of the ileum. Representative flow cytometry results for the ileal mesentery from the control, non-CrF part, and CrF part are shown in Fig. 3A. In terms of the frequency of ILCs in the ileal mesentery, ILC1s were significantly increased in the CrF part, whereas ILC2s and ILC3s were significantly decreased compared to those in the control (Fig. 3B). In terms of the number of ILCs per unit weight of mesentery, the number of ILC1s was significantly increased in the CrF part compared to the control, while the number of ILC2s did not differ between the two groups. Moreover, the number of ILC3s was decreased in the non-CrF part, but did not differ between the CrF part and the control (Fig. 3C). RNA-sequence analysis of CrFs demonstrated that the expression of ILC1-related genes, except *TNFA* and *CCL2*, was upregulated as inflammation intensified (Supplementary Fig. 1E).

Representative flow cytometry results of the ileum from the control, non-CrF part, and CrF part are shown in Supplementary Fig. 2A. In terms of the frequency of ILCs in the ileum, ILC1s were increased and ILC3s were decreased in patients with CD compared to those in the control group, but no difference was found in ILC2s (Supplementary Fig. 2B). Additionally, in terms of the number of ILCs per unit weight of the ileum, the number of ILC1s increased as inflammation intensified, while ILC2s did not differ among the three groups, and ILC3s decreased in the non-CrF part (Supplementary Fig. 2C). Regarding the frequency of ILCs in the mesentery and ileum, a positive correlation was found for all ILCs, suggesting that the degrees of inflammation of the mesentery and ileum are correlated (Fig. 3D, Supplementary Fig. 2D), whereas no positive correlation was found for all ILCs regarding number per unit weight. These results suggest that ILC1s may play an important role in the pathogenesis of CD, as they are increased in both the mesentery and ileum.

### Positive correlation between ILC1s and macrophages in CrF

**Fig. 4:**
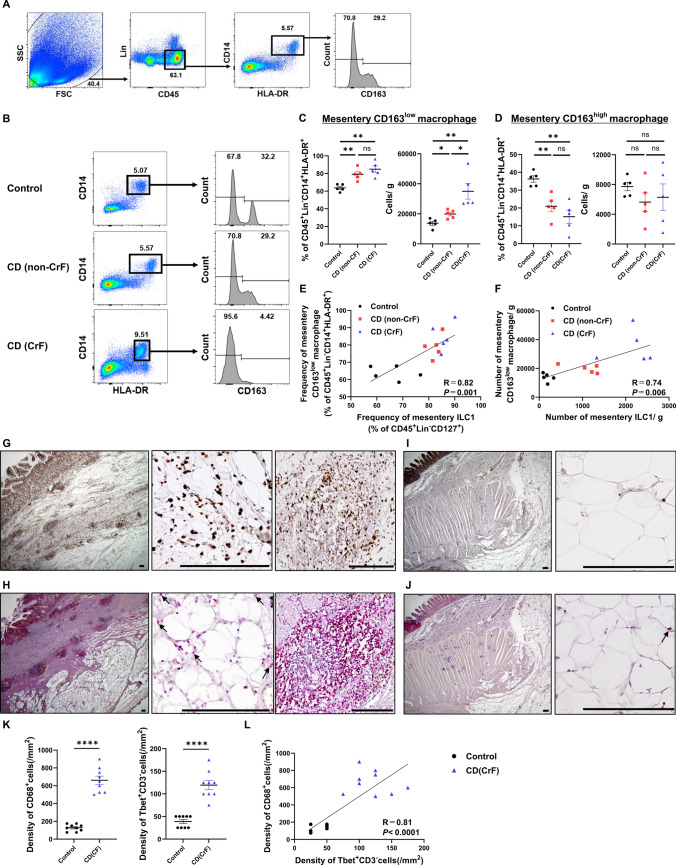
Macrophage subset analysis and immunohistochemistry comparison in human mesentery. **A** Gating of macrophages from human mesentery by flow cytometry. Among CD45^+^ Lin^−^ (CD3^−^ CD19^−^ CD20^−^, CD56^−^) cells, CD14^+^ HLA-DR^+^ cells were defined as macrophages. Macrophages were categorized as CD163^high^ and CD163^low^. **B** Flow cytometric analysis of macrophages from human mesentery. Representative data for five samples each from the control, CD (non-CrF), and CD (CrF) are shown. **C** Altered composition of CD163^low^ macrophage subsets depending on the state of inflammation in human mesentery. Frequencies of mesentery CD163^low^ macrophage subsets among CD45^+^ Lin^−^ CD14^+^ HLA-DR^+^ cells (left) and absolute cell number/g (tissue weight; right) of mesentery. **D** Altered composition of CD163^high^ macrophage subsets depending on the state of inflammation in human mesentery. Frequencies of mesentery CD163^high^ macrophage subsets among CD45^+^ Lin^−^ CD14^+^ HLA-DR^+^ cells (left) and absolute cell number/g (tissue weight; right) of mesentery. Data are presented as mean ± SEM of five independent donors each (*ns* not significant; *P < 0.05, **P < 0.01). **E** Correlation of the frequency between mesentery CD163^low^ macrophage among CD45^+^ Lin^−^ (CD3^−^ CD19^−^ CD20^−^, CD56^−^) CD14^+^ HLA-DR^+^ cells and mesentery ILC1s among 7AAD^−^ CD45^+^ Lin^−^ (CD3^−^ CD11c^−^ CD14^−^ CD16^−^ CD19^−^ CD20^−^) CD127^+^ cells. **F** Correlation of absolute cell number/g (tissue weight) between mesentery CD163^low^ macrophages and mesentery ILC1s. *P* values were obtained by Pearson simple linear regression. **G** Representative images of immunohistochemistry of CrF with CD68 (brown): overview of the intestine and mesentery (left), mesentery (middle), and intestinal attachment area of mesentery (right). **H** Representative images of double immunohistochemistory of CrF with CD3 (brown) and T-bet (red): overview of the intestine and mesentery (left), mesentery (middle), intestinal attachment area of mesentery (right). Arrows indicate CD3^−^ T-bet^+^ cells. **I** Representative images of immunohistochemistory of control mesentery with CD68 (brown); overall view of the intestine and mesentery (left), mesentery (right). **J** Representative images of double immunohistochemistory of the control mesentery with CD3 (brown) and T-bet (red): overall view of the intestine and mesentery (left), mesentery (right). Arrows indicate CD3^−^ T-bet^+^ cells. Scale bars, 200 µm. **K** Comparison of the densities of CD68^+^ cells/mm^2^ (left) and Tbet^+^CD3^−^ cells/mm^2^ (right) between the control mesentery and CrF. Data are presented as mean ± SEM using nine pathological images from three independent donors (****P < 0.0001). **L** Correlation of the density/mm^2^ between CD68^+^ cells and Tbet^+^CD3^−^ cells.* P* values were obtained through Pearson’s simple linear regression analysis

Since macrophages play an important role in adipose tissue fibrosis [17, 18], we investigated the distribution of macrophages in the mesentery and their association with ILC1s, which were increased in CrF. Among the SVFs, CD45^+^ Lin^−^ (CD3^−^ CD19^−^ CD20^−^ CD56^−^) and CD14^+^ HLA-DR^+^ cells were defined as macrophages and divided into CD163^low^ and CD163^high^ macrophages (Fig. 4A). As previously reported in the ileum, CD163^low^ macrophages are inflammatory and are increased in the ileum of patients with CD [14, 19]. Macrophages in the mesentery were examined using the control, CD (non-CrF), and CD (CrF) groups, as shown in Fig. 4B. In the mesentery, the frequency of CD163^low^ macrophages was elevated in CD compared to that in controls, and the number of CD163^low^ macrophages per unit weight (n/g) increased with increasing inflammation (Fig. 4C). By contrast, the frequency of CD163^high^ macrophages was higher in the control group than in the CD group. However, the number of CD163^high^ macrophages per unit weight (n/g) was not significantly different because the overall number of macrophages increased in the CD mesentery (Fig. 4D).

Positive correlations between CD163^low^ macrophage and ILC1s were found for both frequency (*R* = 0.82, *P* = 0.001; Fig. 4E) and number per unit weight (*R* = 0.74; *P* = 0.006; Fig. 4F).

Next, immunostaining for CD68, a macrophage marker, and double immunostaining for CD3, a T cell marker, and T-bet, a marker for both ILC1s and T cells, were performed. CD3^+^ Tbet^+^, CD3^−^Tbet^+^, and CD68^+^ cells were found to be aggregated around adipocytes and at the mesenteric attachment area of CrF (Fig. 4G–H). Several crown-like structures (CLS), a phenomenon in which CD68^+^ cells surround adipocytes, were also observed (Fig. 4G; middle). In CLS there is ongoing inflammation, with CD68^+^ cells forming CLS considered to be the main inflammatory macrophages involved [20]. The images on the right in Fig. 4G and 4H show the clustering of immune cells near the intestinal tract in serial sections, suggesting that CD163^low^ macrophage and ILC1s are present closely together.

Only a small number of immune cells, however, were found in the control mesentery (Fig. 4I–J). Counting CD3^−^Tbet^+^ and CD68^+^ cells and calculating their densities revealed that both cell types were increased in CrF (*P* < 0.0001 for both; Fig. 4K). In addition, a positive correlation was observed between the density of CD3^−^Tbet^+^ and CD68^+^ cells (*R* = 0.81, *P* = 0.001; Fig. 4L). These data suggest a potential interaction between ILC1s and inflammatory macrophages in CrF.

### Fibrogenesis mediated by IFN-γ secreted from ILC1s in CrF

**Fig. 5:**
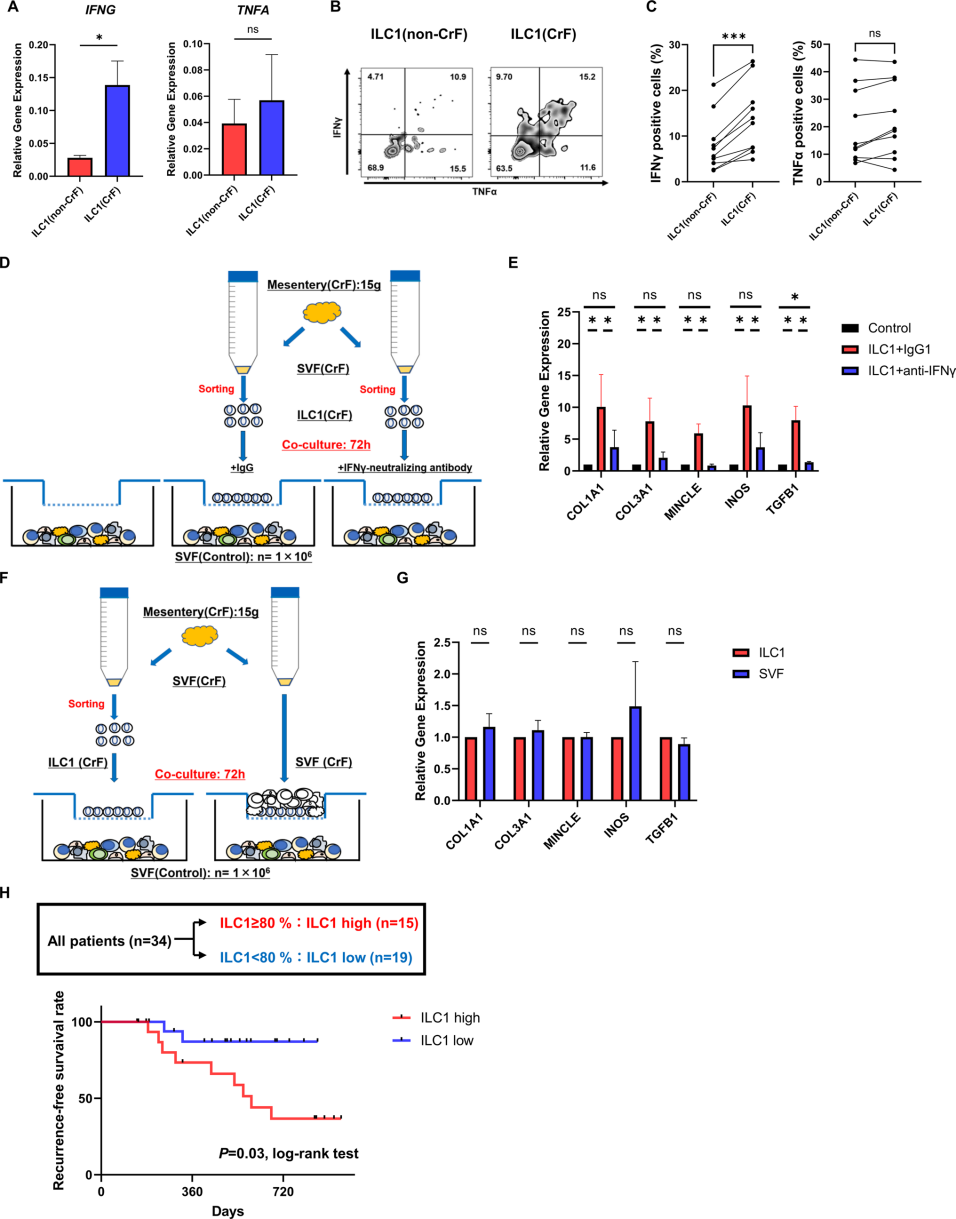
Functional analysis of ILC1s in CrF and clinical impact on Crohn's disease recurrence. **A** Expression of *IFNG* and *TNFA* in ILC1s purified from CrF and non-CrF by qPCR. Data are presented as mean ± SEM of ten independent donors each (ns: not significant; *P < 0.05). **B** Representative images of IFN-γ and TNF-α expression in ILC1 purified from CrF and non-CrF after cell stimulation analyzed by flow cytometry. **C** Expression of IFN-γ and TNF-α in ILC1s purified from CrF and non-CrF by flow cytometry. Data are presented as dots from ten independent donors (ns: not significant; ***P < 0.001). **D** Graphical illustration of co-culture experiments. Viable ILC1s were isolated from 15 g of CrF and cultured in the upper chamber. On the other hand, 1 × 10^6^ of SVFs isolated from the control mesentery were seeded in the lower chamber. Neutralizing IFN-γ antibody was administered during co-culture of SVF from the control with ILC1 from CrF. **E** Relative mRNA expression of *COL1A1*, *COL3A1*, *MINCLE*, *INOS*, and *TGFB1* in human stromal vascular fractions (SVFs) from the control of the lower chamber (*ns* not significant; *P < 0.05). **F** Graphical illustration of co-culture experiments to compare the effects of CrF-derived ILC1s and SVFs on SVFs from the control. Viable ILC1s or SVFs were isolated from 15 g of CrF and cultured in the upper chamber. On the other hand, 1 × 10^6^ of SVFs isolated from the control mesentery were seeded in the lower chamber. CrF-derived SVFs are shown in white. **G** Relative mRNA expression of *COL1A1*, *COL3A1*, *MINCLE*, *INOS*, and *TGFB1* in SVFs from the control cultured in the lower chamber (ns: not significant). **H** Kaplan–Meier estimates demonstrating the percentage of patients with recurrence-free survival after surgery for Crohn’s disease. Patients were divided into two groups: ILC1 high (≥ 80%; *n* = 15) and ILC1 low (< 80%; *n* = 19).* P* values were obtained using the log-rank test

To investigate how ILC1s are involved in CrF formation, we first compared the differences in properties between ILC1s from non-CrF (ILC1 [non-CrF]) and CrF (ILC1 [CrF]) groups, noting that despite the increase in ILC1s in non-CrF group compared to the control, CrF formation was not observed. Regarding ILC1-related cytokines IFN-γ and TNF-α, no differences in TNF-α expression were observed between the two groups, but IFN-γ expression was more prominent in the ILC1 [CrF] group (Fig. 5A–C). However, subgroup analysis revealed an increase in TNF-α-positive cells in ILC1 [CrF] among patients who did not receive anti-TNF-α antibody treatment. This suggests that the majority of patients receiving anti-TNF-α antibodies may have contributed to the absence of changes in TNF-α expression levels within the tissues (*P* = 0.04; Supplementary Fig. 2E). We hypothesized that ILC1-derived IFNγ potentially contributes to the formation of CrFs. Thus, co-culture experiments were subsequently conducted.

Next, to investigate how ILC1s function in the mesentery, SVFs from the control (SVF [control]) were co-cultured with ILC1 [CrF] (Supplementary Fig. 3A). After 72 h, significantly increased expression of fibrosis-related macrophage genes, such as macrophage-inducible c-type lectin (*MINCLE*), inducible nitric oxide synthase (*INOS*), and *TGFB1* were observed in SVF [control] co-cultured with ILC1 [CrF], compared to those cultured without ILC1s (Supplementary Fig. 3B). Moreover, the expression of collagen genes, such as *COL1A1* and *COL3A1*, was also significantly increased in SVF [control] co-cultured with ILC1 [CrF] (Supplementary Fig. 3B).

Next, to evaluate whether IFN-γ from ILC1s promote fibrogenesis of mesentery, neutralizing IFN-γ antibody was administered during co-culture of SVF [control] with ILC1 [CrF] (Fig. 5D). After 72 h of co-culture, *MINCLE*, *INOS*, *TGFB1*, *COL1A1*, and *COL3A1* were significantly increased in the SVFs [control] and ILC1 [CrF] co-culture with IgG isotype control antibody compared with those in the control group. These changes were mostly nullified by administration of neutralizing IFN-γ antibody (Fig. 5E). Since there are other IFN-γ producing cells in the mesentery, such as T and NK cells, further co-culture experiments were conducted to determine whether ILC1s are essential for fibrogenesis by comparing the effects of CrF-derived ILC1s and SVFs on SVFs from the control (Fig. 5F). There was no difference in fibrosis-related gene expression in SVFs from the control compared to that in ILC1s and SVFs from CrF (Fig. 5G). These findings indicate that IFN-γ secreted from ILC1s is essential for the fibrogenesis in CrF.

### Contribution of elevated mesenteric ILC1s to endoscopic recurrence in patients with CD

Next, we investigated the clinical importance of mesenteric ILC1s. Thirty-four patients with CD who underwent postoperative endoscopic examination after bowel resection were divided into two groups, one with recurrence (n = 11) and the other without recurrence (n = 23), and risk factors were assessed. As shown in Table 2, a high frequency of ILC1s in the CrF mesentery (*P* = 0.01) was a risk factor for early endoscopic recurrence. Conversely, there were no differences in preoperative or postoperative treatment between the two groups. Furthermore, multivariate analysis was performed, encompassing age, body mass index (BMI), preoperative serum CRP levels, and preoperative serum albumin levels, and a high frequency of ILC1 in the CrF mesentery was confirmed to be an independent risk factor (*P* = 0.01, odds ratio: 1.25). Next, ROC curve analysis identified a cutoff value of 80 for ILC1s in the CrF mesentery (%), and the patients with CD were divided into two approximately even groups—ILC1 high (≥ 80%; n = 15) and ILC1 low (< 80%; n = 19)—and a recurrence-free survival curve was drawn. At the median follow-up period of 520 days, the log-rank test analysis showed a significantly higher recurrence rate in the ILC1 high group (*P* = 0.03; Fig. 5H).

**Table 2:** Risk factors for postoperative recurrence of Crohn's disease

	Recurrence group (*n*=11)	Non-recurrence group (*n*=23)	*P*-value	Multivariate analysis	
				*P*-value	Odds ratio (95% CI)
Age at surgery, years	35.7 ± 11.4	36.8 ± 14.7	0.77	0.47	0.97 (0.87–1.07)
Sex, male/female	9 (81.8%)/2 (18.2%)	17 (73.9%)/6 (26.1%)	0.25		
Body mass index, kg/m^2^	19.2 ± 1.9	19.2 ± 3.1	0.98	0.29	0.71 (0.36–1.39)
Disease duration, years	11.1 ± 7.9	10.1 ± 10.1	0.77		
Smoking	2 (18.2%)	3 (12.5%)	0.69		
Surgical indication					
Stenosis Fistula formation	9 (81.8%) 2 (18.2%)	16 (69.6%) 7 (30.4%)	0.44		
			0.94		
Past history of intestinal resection	0 (0.0%)	3 (12.5%)	0.74		
Preoperative medication*					
Steroid	3 (27.3%)	3 (12.5%)	0.31		
5-ASA	8 (72.7%)	20 (83.3%)	0.31		
Anti-TNFα antibody	6 (54.5%)	13 (54.2%)	0.91		
Preoperative serum CRP, mg/L	1.2 ± 1.5	0.8 ± 1.8	0.47	0.93	0.98 (0.56–1.70)
Preoperative serum Albumin, g/dL	3.2 ± 0.7	3.6 ± 0.5	0.08	0.13	0.07 (0.002–2.23)
Frequency of ILC1 in the mesentery of CF part**, %	84.9 ± 6.0	77.8 ± 7.5	0.01	0.02	1.25 (1.03–1.51)
Postoperative medication*					
Steroid	0 (0%)	0 (0%)	1.00		
5-ASA	8 (72.7%)	20 (83.3%)	0.31		
Anti-TNFα antibody	7 (63.6%)	15 (65.2%)	0.93		

Data are presented as *n* (%) or mean ± SD. *5-ASA* 5-aminosalicylic acid, *TNF* tumor necrosis factor, *CRP* C-reactive protein, *ILC* innate lymphoid cell, *CF* creeping fat. *, included duplicate; **, frequency of ILC1 among CD45^+^Lin^−^ CD127^+^ cells

## Discussion

CD research has primarily focused on the gastrointestinal tract as the initiation site of symptoms and exacerbation. Pathological studies on mucosal immunity and gut microbiota have increased; however, studies on CrF remain limited. Our study revealed increased ILC1s in CrF with high IFN-γ production, correlating with inflammatory macrophages, even without macroscopic inflammation in the mesentery of patients with CD.

Mesenteric inflammation is anatomically linked to transmural inflammation and mucosal ulceration, and correlates with intestinal inflammation severity on histological examination [5, 8]. CrF is an expansion of mesenteric adipose tissue surrounding the intestinal wall [3]. During embryological development, the intestinal epithelium is derived from the endoderm, which is surrounded by the mesoderm that forms the mesenchyme and mesentery [21]. Mesenchymal connective tissue and mesentery maintain continuity until adulthood, which is important in CD pathogenesis [21]. In our study, CrF formation in the mesentery was consistent with transmural inflammation. In CD, the boundary between the mesentery and intestine is blurred, with ECM scaffold thickening between the serosa and muscularis propria, and direct contact between adipocytes and smooth muscle cells [22]. Connective tissue thickening was also observed in our study, along with edematous mesentery, dense infiltration of inflammatory cells, atrophic adipocytes, and increased collagen. These results indicate extraintestinal-to-luminal inflammation and fibrosis.

The SVF of the mesentery contains diverse cells important for adipose function. While adipocytes occupy over 90% of fat pad volume, there are 1–2 million adipocytes per gram of adipose tissue compared to 4–6 million per gram of SVF cells (including endothelial cells, immune cells, fibroblasts, preadipocytes, and stem cells) [23]. In mice, ILC1s account for approximately 22–30% of lymphocytes in adipose tissue and constitute the most abundant lymphocyte population [24, 25]. We found that all three ILC subsets (ILC1, ILC2, and ILC3) in the mesentery of both the CD and control groups. ILC1s were more abundant in the CD group, especially in the CrF. The overall ILC count was increased in CrF, with even ILC2 and ILC3 maintaining a higher count, despite decreased frequency. This aligns with previous reports of increased immune-related lymphocytes in CrF [6, 26]. Additionally, we observed a decrease in ILC3s in the non-CrF parts, but a recovery in the CrF parts compared to those in the control. In the blurred interface of CrF, we observed clusters of lymphocytes, such as tertiary lymphoid organs (TLOs), previously reported to consist of B cells and apparent ILCs that had invaded the lymphatic vessel wall [27]. The ILC3 recovery may result from TLO formation by lymphoid tissue inducer cells—a subset of ILC3—in response to bacterial translocation from the intestinal lumen to the mesentery [13].

We found that increased expression of both ILC1- and macrophage-associated genes were involved in CrF fibrosis, other than those for TNF-α and CCL2. Mesenteric ILC1s and macrophages were positively correlated in frequency and number of cells per unit weight. Previous reports show that under high-fat-diet challenges, ILC1s in adipose tissue are increased and activated as a major source of IFN-γ and TNF- α. ILC1s induce adipose tissue macrophages to M1 polarization, leading to adipose tissue inflammation and insulin resistance [12]. IFN-γ produced by ILC1 triggers macrophage activation, defending the host against intracellular parasitic bacteria such as *Toxoplasma gondii* [28]. In high-fat-fed obese mice models, ILC1s accumulate and produce IFN-γ in subcutaneous adipose tissue, directly inducing local inflammation and fibrogenesis by stimulating inflammatory macrophages to secrete TGF-β1 and activate the TGF-β1/smad3 signaling pathway [12]. In this study, IFN-γ was significantly elevated in CrF, indicating that CrF inflammation is activated through ILC1 and macrophage interaction via INF-γ. However, TNF-α and CCL2 were not elevated in CrF. ILC1-derived CCL2 is a macrophage attractant and key regulator of ILC1-macrophage signaling [29], while TNF-α induces CCL2 in adipose tissue [30]. Despite inflammation in CrF, most of our samples were from patients receiving anti-TNF-α drugs, which may have suppressed TNF-α and CCL2 expression.

Despite reports of increased macrophages in CrF, whether inflammatory or non-inflammatory macrophages are predominant remains controversial. Recent reports show that *Clostridium innocuum* translocates within CrF, stimulating tissue remodeling through CD206^+^ non-inflammatory macrophages, while other CD-specific gut bacteria induce inflammatory macrophages [31]. Moreover, genes associated with microbial sensing and killing are highly expressed in CrF [7]. In this study, in mesenteric macrophages, CD163^low^ cells per unit weight were increased in inflammatory areas, and there was a positive correlation between the number of ILC1 and CD163^low^ cells. This suggests that during CrF formation in the mesentery, adipose tissue prevents intestinal injury and bacterial translocation and hinders systemic antigen exposure, but changes aggressively in the uncontrolled inflammatory phase. Regarding macrophage inflammatory/anti-inflammatory polarization, potential influencing factors include surgical indication and timing, race, BMI, and age.

In our previous study, we found that the number of CD163^low^ cells was increased in the inflammatory areas of the intestinal tract similar to the present results in the CD mesentery. These cells express TLR2, TLR4, and TLR5, induce IL-6 and IL-23, and promote Th1/17 cell-mediated immunity [14]. Under homeostatic conditions, CD163^high^ macrophages are the primary producers of TGF-β; however, in inflamed intestinal tissues, CD163^low^ macrophages also produce significant TGF-β [14]. Furthermore, intestinal CD163^high^ macrophages can be subdivided into CD160^high^ and CD160^low^ populations. CD160^high^ macrophages can suppress T-cell proliferation but are reduced in inflamed intestinal tissues, leading to their immunosuppressive function loss. Meanwhile, CD160^low^ macrophages—the main producers of IL-10 under homeostatic conditions—increase in inflamed intestines and shift toward inflammatory cytokine production such as IL-6 [32].

CLS are a pathological finding in which macrophages surround and phagocytose dysfunctional adipocytes, indicating the site of cell–cell interaction and the origin of chronic inflammation [18]. Our pathological examinations showed increased CLS, adipocyte atrophy, and fibrogenesis in CrFs compared with those in the control. Adipocytes, which secrete biologically active substances known as adipokines, exhibit endocrine or paracrine functions and regulate local and systemic homeostasis [33, 34]. However, in CD mesentery, they are involved in CrF formation and fibrosis through interactions with neighboring cells. Recently, TLR4-mediated macrophages were shown to play an important role in the aberrant remodeling of the ECM, affecting adipocyte dysfunction in CrF [18, 35]. Our previous study identified CD163^low^ cells as TLR4-expressing macrophages involved in profibrogenic function through nitric oxide production and Mincle expression [12, 14, 17]. Fibrosis-related genes such as *MINCLE, INOS, TGFB1, COL1A,* and *COL3A* were significantly increased in control SVFs co-cultured with CrF-derived ILC1s compared to control SVFs cultured without ILC1s. These effects were cancelled by administering neutralizing IFN-γ antibody. These results suggest that ILC1s contribute to the macrophage-associated fibrogenesis of mesentery through IFN-γ production.

Until recently, CD surgery has focused on preserving the bowel as much as possible by avoiding mesentery resection [36]. This may be attributed to the high redo surgery rates because of disease characteristics and repeated intestinal resection possibly resulting in intestinal failure, causing the patient to require nutrient intake through the parenteral route. Following reports on the clinical impact of CrF, patients with CD undergoing ileocolic resection with mesentery dissection, similar to oncological techniques, showed a cumulative reoperation rate of only 2.9%, compared to 40% in those without routine CrF resection [22]. However, mesentery dissection is not always feasible. Since the mesentery of patients with CD is characteristically thickened and well vascularized, its division is particularly difficult and is sometimes accompanied by massive bleeding. Our study revealed that postoperative endoscopic recurrence was higher in patients with more ILC1 in the CrF. Therefore, controlling the CrF may present a novel strategy for improving CD outcomes.

This study has several limitations. We focused on the role of ILC1 and macrophages in CrF; however, the pathology of the CD mesentery may be multifactorial. Our results may represent only one factor and could not exclude other cells and mechanisms involved in the pathology, such as mast cells, eosinophils, lymphocytes, and stromal cells. Additionally, factors such as pre- and postoperative medical treatments, surgical indications, and patient characteristics—including race, body type, and age—were not accounted for, and their potential influence on the results cannot be overlooked. Furthermore, this study confirmed the association between ILC1 and postoperative recurrence in resected CrFs. However, it did not investigate the relationship between ILC1 and recurrence in the remaining intestinal tract or its mesentery. Consequently, the association between systemic ILC1 and postoperative recurrence warrants further investigation.

## Conclusions

Our study demonstrated an increase in ILC1s in CD-affected mesentery, and their role in fibrogenesis through IFN-γ and macrophages. Notably, ILC1s were elevated even without inflammation, indicating their involvement in CrF formation and potential effect on the intestinal tract. These findings suggest that treatments must target both the intestinal mucosa and mesentery in patients with CD. Future studies should explore the contribution of other immune cells, their interactions with ILC1s and macrophages, and preoperative factors that influence CrF development and disease progression. Prospective studies with larger populations could reveal the impact of patient diversity, aiding personalized treatment.

## Supplementary Information

Below is the link to the electronic supplementary material.

Supplementary file1 (TIF 156618 KB)

Supplementary file2 (TIF 150142 KB)

Supplementary file3 (TIF 37697 KB)

Supplementary file4 (DOCX 20 KB)

Supplementary file5 (DOCX 23 KB)

Supplementary file6 (DOCX 16 KB)

Supplementary file7 (DOCX 16 KB)
