# Supplementary figures and images for: Group 1 innate lymphoid cells and inflammatory macrophages exacerbate fibrosis in creeping fat through IFN-γ secretion
Source: J Gastroenterol. 2025 Mar 29;60(7):838–53. doi: 10.1007/s00535-025-02243-x (PMC12176962; doi:10.1007/s00535-025-02243-x)

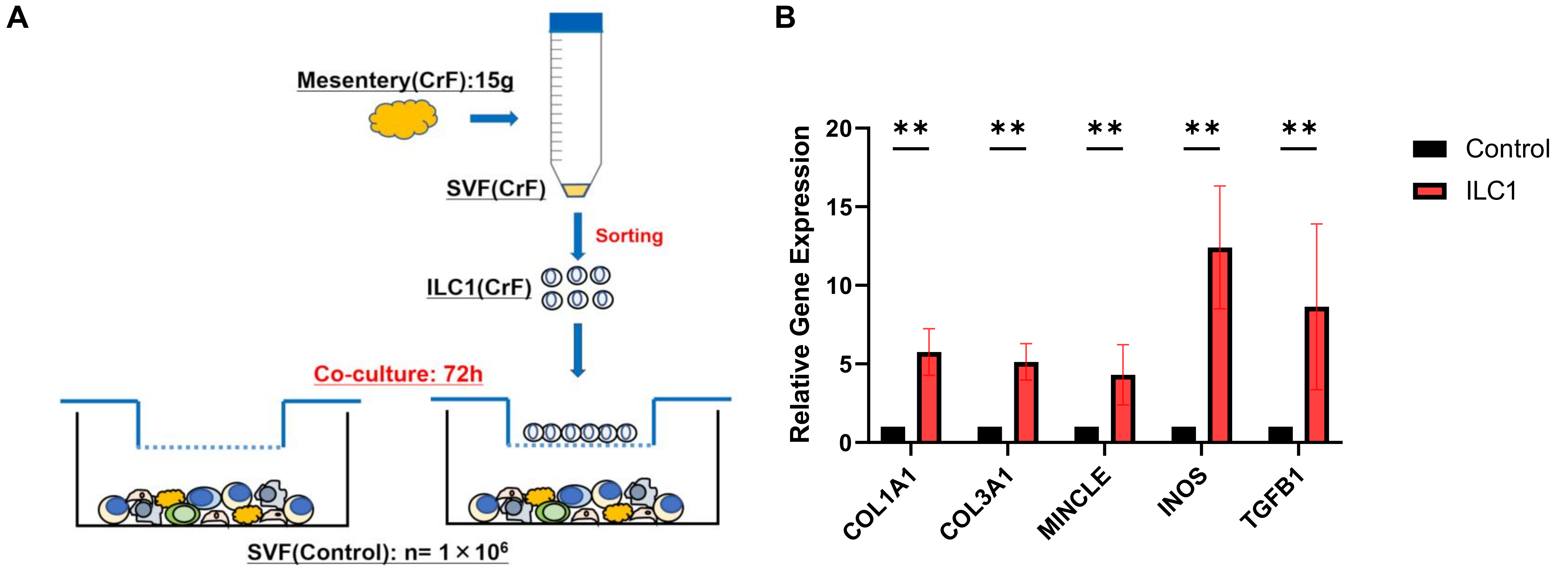

Supplement: Supplementary file 3 — Supplementary file3 (TIF 37697 KB) [file 535_2025_2243_MOESM3_ESM.tif]
