# Supplementary material for: Group 1 innate lymphoid cells and inflammatory macrophages exacerbate fibrosis in creeping fat through IFN-γ secretion
Source: J Gastroenterol. 2025 Mar 29;60(7):838–53. doi: 10.1007/s00535-025-02243-x (PMC12176962; doi:10.1007/s00535-025-02243-x)
Supplement: Supplementary file 4 — Supplementary file4 (DOCX 20 KB) [file 535_2025_2243_MOESM4_ESM.docx]

**Supplementary methods**

**RNA sequencing:**

Total RNA was obtained from the CrF and non-CrF parts of the ileal mesentery of 20 patients with Crohn’s disease and 10 controls for RNA sequence analysis. We used the miRNeasy Mini Kit (Qiagen, Hilden, Germany) to extract total RNA from the bulk sample of the ileal mesentery of individual patients and mixed it in equal amounts, following the manufacturer’s instructions. A library was prepared using a TruSeq stranded mRNA sample prep kit (Illumina, San Diego, CA, United States), according to the manufacturer's instructions. Whole-transcriptome sequencing was performed on the RNA samples using the NovaSeq 6000 Platform (Illumina) in 101-base single-end mode. Sequenced reads were mapped onto mouse reference genome sequences (mm10) using TopHat ver. 2.1.1. The number of fragments per kilobase of exons per million mapped fragments was calculated using Cufflinks ver. 2.2.1. The extracted transcript reads were normalized to the study area and uploaded to IDEP, a web tool for visualizing the clustering of multivariate data using heat maps and K-means analysis.

**Supplementary Figure 1**. [A] K-means cluster analysis of the differentially expressed genes in the mesenteric adipose tissue based on RNA sequencing. Genes were classified into four clusters according to the site from which the samples were obtained and the degree of gene expression. [B] Comparison of the long diameter of adipocytes in the control and creeping fat (CrF). Ten adipocytes were randomly selected from four independent donors. Data are presented as mean ± standard error of the mean (SEM) (*****P <* 0.0001). [C] Heat map representation of fibrosis-related gene expression profiles based on RNA-sequence analysis of a bulk sample of the mesentery. Ten samples were obtained and mixed in equal amounts for the analysis. [D] Flow cytometric analysis of innate lymphoid cell (ILC) subsets from human fat tissues. Representative data for the subcutaneous fat, omentum, and mesentery of the colon are shown. [E] Heat map of relative ILC1-related gene expression profiles based on the RNA-sequence analysis of bulk mesentery samples. Ten samples were obtained and mixed in equal amounts for the analysis.

**Supplementary Figure 2.** [A] Flow cytometric analysis of ILC subsets from human ileum. Representative data from control, non-CrF, and CrF groups are shown. [B] Frequency of ILC subsets in 7AAD^-^ CD45^+^ CD127^+^ Lin^-^ (CD3^-^ CD11c^-^ CD14^-^ CD16^-^ CD19^-^ CD20^-^) cells depending on the state of inflammation in the human ileum. [C] Absolute cell number/g (tissue weight) of ILC subsets depending on the state of inflammation in the ileum. Data are presented as mean ± SEM of 10 control donors and 13 Crohn's disease donors (ns: not significant, **P <* 0.05, ****P <* 0.001, *****P <* 0.0001). [D] Correlation of frequency and absolute cell number/g (tissue weight) of ILC2 and ILC3 between the ileum and mesentery. *P*-values were obtained through Pearson’s simple linear regression analysis. [E] Expression of IFN-γ and TNF-α in ILC1s purified from CrF and non-CrF samples, analyzed by flow cytometry and stratified based on preoperative use of anti-TNF-α antibodies. The group that received preoperative anti-TNF-α antibodies is shown on the left (N = 6) and the group that did not receive them is shown on the right (N = 4). Data are presented as individual dots representing independent donors (ns: not significant; *P < 0.05).

**Supplementary Figure 3.** [A] Graphical illustration of the co-culture experiments. Viable ILC1s were isolated from 15 g of CrF and cultured in the upper chamber. On the other hand, 1 × 10^6^ of SVFs isolated from the control mesentery were seeded in the lower chamber. [B] Relative mRNA expression levels of *COL1A1*, *COL3A1*, *MINCLE*, *INOS*, and *TGFB1* in human stromal vascular fractions from the control cultured in the lower chamber (ns: not significant, ***P <* 0.01).
