# Supplementary material for: Group 1 innate lymphoid cells and inflammatory macrophages exacerbate fibrosis in creeping fat through IFN-γ secretion
Source: J Gastroenterol. 2025 Mar 29;60(7):838–53. doi: 10.1007/s00535-025-02243-x (PMC12176962; doi:10.1007/s00535-025-02243-x)
Supplement: Supplementary file 5 — Supplementary file5 (DOCX 23 KB) [file 535_2025_2243_MOESM5_ESM.docx]

**Supplementary Table S1. Patient characteristics**

| Analyzed area | | RNA-seq | Age | Sex | Surgical indication | Duration | Montreal classification | | | Body mass index | Preoperative medication | | |
| --- | --- | --- | --- | --- | --- | --- | --- | --- | --- | --- | --- | --- | --- |
| Ileum | Mesentery |  |  |  |  |  | A | L | B |  | 5-ASA | Steroid | Anti TNF-α |
| ○ | ○ | ○ | 33 | Female | Stenosis | 4 | 2 | 3 | 2 | 20.3 | ○ |  | ○ |
| ○ | ○ | ○ | 33 | Male | Stenosis | 12 | 2 | 1 | 2 | 18.8 | ○ | ○ | ○ |
| ○ | ○ | ○ | 20 | Male | Stenosis | 4 | 1 | 1 | 2 | 17.9 | ○ |  | ○ |
| ○ | ○ | ○ | 24 | Male | Fistula | 1 | 2 | 3 | 3 | 20.8 | ○ |  | ○ |
| ○ | ○ | ○ | 23 | Male | Stenosis | 8 | 1 | 3 | 2 | 19.9 | ○ |  | ○ |
| ○ | ○ | ○ | 53 | Male | Fistula | 22 | 2 | 1 | 3 | 22.7 | ○ |  | ○ |
| ○ | ○ | ○ | 36 | Female | Stenosis | 7 | 2 | 3 | 2 | 20.2 | ○ |  | ○ |
| ○ | ○ | ○ | 21 | Male | Stenosis | 5 | 3 | 3 | 2 | 21.8 | ○ | ○ | ○ |
| ○ | ○ | ○ | 41 | Male | Fistula | 19 | 2 | 3 | 3 | 22.2 |  |  |  |
| ○ | ○ | ○ | 25 | Male | Stenosis | 7 | 2 | 3 | 2 | 14.5 | ○ |  | ○ |
| ○ | ○ | ○ | 74 | Male | Stenosis | 6 | 2 | 1 | 2 | 18.0 | ○ |  |  |
| ○ | ○ | ○ | 35 | Male | Fistula | 0 | 2 | 1 | 3 | 17.5 |  |  |  |
| ○ | ○ | ○ | 19 | Male | Stenosis | 0 | 2 | 1 | 2 | 18.5 | ○ |  | ○ |
|  | ○ | ○ | 39 | Male | Stenosis | 16 | 2 | 3 | 2 | 20.0 | ○ |  |  |
|  | ○ |  | 47 | Male | Stenosis | 17 | 2 | 1 | 2 | 23.8 | ○ |  |  |
|  | ○ |  | 17 | Male | Stenosis | 2 | 1 | 3 | 2 | 17.3 | ○ |  |  |
|  | ○ |  | 47 | Male | Stenosis | 21 | 2 | 1 | 2 | 17.1 |  |  |  |
|  | ○ | ○ | 38 | Male | Stenosis | 11 | 2 | 3 | 2 | 16.6 | ○ |  | ○ |
|  | ○ | ○ | 21 | Male | Stenosis | 2 | 2 | 3 | 2 | 16.8 | ○ | ○ |  |
|  | ○ |  | 48 | Male | Fistula | 20 | 2 | 1 | 3 | 14.6 | ○ |  |  |
|  | ○ |  | 21 | Male | Stenosis | 4 | 2 | 3 | 2 | 19.4 | ○ |  |  |
|  | ○ | ○ | 47 | Male | Stenosis | 2 | 2 | 3 | 2 | 19.5 | ○ | ○ | ○ |
|  | ○ |  | 48 | Female | Fistula | 18 | 2 | 3 | 3 | 22.6 | ○ |  |  |
|  | ○ | ○ | 35 | Male | Stenosis | 25 | 1 | 1 | 2 | 17.4 | ○ |  | ○ |
|  | ○ |  | 28 | Female | Stenosis | 13 | 1 | 1 | 2 | 14.9 | ○ |  |  |
|  | ○ | ○ | 50 | Male | Stenosis | 24 | 2 | 3 | 2 | 21.0 | ○ |  | ○ |
|  | ○ |  | 64 | Male | Stenosis | 39 | 2 | 1 | 2 | 21.9 |  |  |  |
|  | ○ |  | 34 | Female | Stenosis | 2 | 2 | 3 | 2 | 17.7 | ○ | ○ | ○ |
|  | ○ | ○ | 40 | Male | Fistula | 9 | 2 | 3 | 3 | 17.6 | ○ |  | ○ |
|  | ○ |  | 31 | Male | Stenosis | 1 | 2 | 1 | 2 | 22.0 |  |  |  |
|  | ○ |  | 43 | Female | Stenosis | 3 | 2 | 3 | 2 | 26.5 | ○ |  | ○ |
|  | ○ |  | 39 | Female | Fistula | 6 | 2 | 3 | 3 | 17.5 |  |  | ○ |
|  | ○ |  | 47 | Male | Stenosis | 21 | 2 | 1 | 2 | 16.3 | ○ |  | ○ |
|  | ○ |  | 18 | Male | Fistula | 3 | 1 | 3 | 3 | 19.5 | ○ | ○ |  |
| 5-ASA, 5-aminosalicylic acid; TNF, tumor necrosis factor | | | | | | | | | | | | | |
