# Supplementary material for: Group 1 innate lymphoid cells and inflammatory macrophages exacerbate fibrosis in creeping fat through IFN-γ secretion
Source: J Gastroenterol. 2025 Mar 29;60(7):838–53. doi: 10.1007/s00535-025-02243-x (PMC12176962; doi:10.1007/s00535-025-02243-x)
Supplement: Supplementary file 6 — Supplementary file6 (DOCX 16 KB) [file 535_2025_2243_MOESM6_ESM.docx]

**Supplementary Table S2. Anti-human antibodies used for flow cytometry**

| CD3-FITC | HIT3a | BioLegend, CA, USA |
| --- | --- | --- |
| CD11c-FITC | Bu15 | BioLegend, CA, USA |
| CD11c-APC | SHCL-3 | BD Biosciences, CA, USA |
| CD14-FITC | HCD14 | BioLegend, CA, USA |
| CD14-PE | HCD14 | BioLegend, CA, USA |
| CD19-FITC | HIB19 | BioLegend, CA, USA |
| CD20-FITC | 2H7 | BioLegend, CA, USA |
| CD56-FITC | HCD56 | BioLegend, CA, USA |
| CD45-APC/Cy7 | HI30 | BioLegend, CA, USA |
| CD117-PE | 104D2 | BioLegend, CA, USA |
| CD127-BV421 | M21 | BD Biosciences, CA, USA |
| CD163-PerCP/Cy5.5 | GHI/61 | BioLegend, CA, USA |
| CRTH2-PE | BM16 | Miltenyi Biotec, Bergisch Gladbach, Germany |
| CRTH2-FITC | BM16 | BioLegend, CA, USA |
| HLA-DR-PE/Cy7 | SHCL-3 | BioLegend, CA, USA |
| T-bet-PE | 4B19 | eBioscience, CA, USA |
| ROR gamma t-PE | AFKJS-9 | eBioscience, CA, USA |
| IFN-γ-PE | B27 | BioLegend, CA, USA |
| TNF-α-APC | MAb11 | BioLegend, CA, USA |
