# Supplementary material for: Group 1 innate lymphoid cells and inflammatory macrophages exacerbate fibrosis in creeping fat through IFN-γ secretion
Source: J Gastroenterol. 2025 Mar 29;60(7):838–53. doi: 10.1007/s00535-025-02243-x (PMC12176962; doi:10.1007/s00535-025-02243-x)
Supplement: Supplementary file 7 — Supplementary file7 (DOCX 16 KB) [file 535_2025_2243_MOESM7_ESM.docx]

|  | F | R |
| --- | --- | --- |
| *GAPDH* | GTCGGAGTCAACGGATT | AAGCTTCCCGTTCTCAG |
| *COL1A1* | GAGGGCCAAGACGAAGAATC | GAGGGCCAAGACGAAGACATC |
| *COL3A1* | GGAGCTGGCTACTTCTCGC | GGGAACATCCTCCTTCAACAG |
| *MINCLE* | AAGAACTGCTCAGCCATGGG | CCTGCTCCTCCTGTGAGTTGA |
| *INOS* | TTCAGTATCACAACCTCAGCAAG | TGGACCTGCAAGTTAAAATCCC |
| *IFNG* | CCAGGACCCATATGTAAAAG | TGGCTCTGCATTATTTTTC |
| *RORC* | CCCGTCAGCAGAACTG | AGCCCCAAGGTGTAGG |
| *TBX21* | GATGTTTGTGGACGTGGTCTTG | CTTTCCACACTGCACCCACTT |
| *TGFB1* | TGGAAGTGGATCCACGCGCCCAAGG | GCAGGAGCGCACGATCATGTTGGAC |
| *TNFA* | CGCTCCCCAAGAAGAC | AGGGCTGATTAGAGAGAGGT |

**Supplementary Table S3. Primer sets used for polymerase chain reaction**
